# Progress in Surgery

**Published:** 1896-06-20

**Authors:** 


### Progress in Surgery.

### DISEASES OF THE ABDOMEN.

**Pancreas.**—A case of pancreatitis with hæmorrhage that occurred under the care of Dr. Rolleston is recorded.[1] The pancreas presented a curious appearance. Although the lobules were distinct, the gland was infiltrated with putty-like material, situated apparently in the interstial tissue. A layer of similar substance covered the surface of the gland. Over this again was a layer of blood about three-eighths of an inch thick, which spread outwards on either side over the surface of both kidneys and the suprarenal bodies. The posterior surface of the stomach was covered with recent soft lymph, and behind it, in the cavity of the omentum, were several ounces of a fluid like milk. There were also spots of fat necrosis in the mesentery, great omentum, and fat of the abdominal wall. The case occurred in a man aged thirty-one, who was seized with severe pain in the abdomen while at work. This passed off after having been present for an hour and a half, but recurred soon after a midday meal of roast mutton. The pain persisted until the evening, and kept him awake at night. He vomited once the following morning. The bowels acted shortly before the onset of the pain, but had not again at the time of admission to St. George's Hospital, about twenty-six hours later. At that time, in appearance he was not thought to give the impression of serious illness. There was slight distension of the abdomen in the epigastrium, with some localised tenderness. The abdomen moved well with respiration. He complained of pain below the ribs in the umbilical region and in the small of the back. At six p.m. on the day of admission the temperature was 103 deg., but later he became markedly collapsed, and the temperature fell to 95 deg. The pulse remained quick—132. The collapse continued throughout the following day, he became delirious, his temperature again rose to 103 deg., and he died about sixty hours after the first attack of abdominal pain. The question of diagnosis between acute intestinal obstruction and acute peritonitis following perforation of the stomach or appendix is discussed. It is thought that the absence of continued vomiting and of abdominal distension would, in another case, perhaps, suggest pancreatitis rather than intestinal obstruction or peritonitis. Riedel[2] describes inflammatory enlargements of the head of the pancreas that may be mistaken for growth. They occur in cases of gall-stone, in which possibly a calculus has been impacted from time to time at the duodenal end of the common bile duct. Riedel's attention was first attracted to the condition in a patient on whom he operated for gall-stones. A gall-stone was found in the gall-bladder, and the cystic and common bile ducts were widely dilated, but contained no stones. A tumour the size of a small apple was felt in the head of the pancreas. It was very hard, and thought to be carcinomatous in nature. The patient, however, improved rapidly after the operation, and two and a quarter years after the operation was quite well. Two other similar cases were met with, both at the time of operation for gall-stones. One patient improved, and in a year and a half gained 30 lbs. in weight; the other died, and after death an interstitial inflammation of the pancreas was found, but no signs of malignant growth. Stiller[3] says that cancer of the pancreas is not so uncommon as is generally supposed. Segré is quoted as saying that in 627 cases of abdominal cancer the pancreas was involved in no less than 127. In only 12, however, the disease was limited to the pancreas. The diagnosis is referred to as difficult, since a tumour can rarely be felt. Stiller considers, however, that marked dyspeptic symptoms, such as anorexia, vomiting, pain, where there is absence of dilatation of the stomach, but rapid wasting, subnormal temperature, and later progressive and persistent jaundice without enlargement of the liver, are sufficient to warrant the supposition of cancer of the pancreas. In rare instances glycosuria, fatty motions, salivation, and bronzing of the skin may be present.

**Spleen.**—Schäfer and Moore[4] write on the rhythmic contractility of the spleen and the effect of animal extracts upon it. The rhythmic contractions are independent of the nervous system, since they go on after all the nerves have been severed. Stimulation of the branches of the splanchnics, however, which accompany the arteries entering the spleen, causes strong contraction of the organ. Apparently there are also nerve fibres running in the splanchnics that cause dilatation of the spleen. The vagus does not appear to have the power of affecting the volume of the spleen. Dyspnœa causes marked contraction of the spleen and apparently through the nervous system, since after severance of the nerves the gland dilates. The rhythmic contractions, however, go on, and are increased in extent. Certain drugs and animal extracts also have considerable influence on the contractions of the spleen. Curare and brain extract cause great increase of the rhythmic contraction. Suprarenal extract has the same effect after a preliminary contraction described as enormous. A successful removal of a wandering spleen is recorded by Runge,[5] and Kouwer[6] publishes two cases of the same disease treated by stitching the gland to the abdominal wall. Both cases were in women in whom the displaced spleen seemed to have given rise to distressing symptoms. In one the operation was permanently successful, and the distressing symptoms disappeared.

**Suprarenal Capsules.**—Osler[7] records six cases of Addison's disease and one in which suprarenal extract had been given for eight months. The patient, a man aged forty-six years, was admitted to the Johns Hopkins Hospital with the characteristic bronzing of Addison's disease and, in addition, signs of phthisis at the right apex. There were also prostration and rapid heart action, considered to be greater in amount than the lung disease would account for. A fortnight after admission treatment by suprarenal extract, prepared from the fresh suprarenal bodies of pigs, was commenced. At the end of three weeks he was taking the equivalent of three glands daily. In four months he had gained nineteen pounds, and he was bright and active, and he left the hospital. Some four months later he was still in good health, and said that he felt as well as he ever had done in his life. The condition of his lung had considerably improved. The pigmentation, however, remained much the same.

**Intestines.**—Tull Walsh[8] writes on the treatment of dysentery. He says that his experience of ipecacuanha sine emetina has not been favourable. He thinks that emetin is the active principle that is of value in the cure of dysentery. Acting on this belief he used a compound of emetin with $HgI_2$. This compound remains combined in the presence of acids, and does not produce vomiting when introduced into the stomach. There is, in addition, the valuable antiseptic action of the $HgI_2$. Of thirty-nine cases treated only one died in the hospital, and one went out before a cure could have been said to have been completed. The rest recovered. In most cases nearly one grain of emetin was taken daily. The writer, however, goes on to say that in all probability the value of ipecacuanha has been over-rated. Cases treated with holanhena, with tincture of aconite and rice and milk diet, with naphthalin, or with sulphuric acid and sulphate of magnesia, have given almost, if not quite, as good results as ipecacuanha. Tull Walsh concludes, therefore, that too great stress should not be laid upon any particular drug. Treatment founded upon common-sense principles should mainly be followed. Cleanliness of the large intestine should be maintained by mild purgatives and rest by fluid diet. Hæmorrhage, when severe, should be treated, and hazeline is considered the best remedy. Pain may also require treatment by morphia, but it is said to generally pass off when the intestine is cleared of fæces.

Chauffard[9] describes a case in which the presence of ascarides seems to have given rise to symptoms that simulated those of typhoid fever. There was some pyrexia associated with lassitude, headache, sleeplessness, ileo-cæcal gurgling, tenderness of the abdomen, and slight enlargement of the spleen. He repeatedly passed round worms in the fæces, and vomited one. He was given santonin, which brought away eleven worms one day and four the next. From time to time fresh santonin was given, always with the result that ascarides came away. He left the hospital against advice before his fæces were free from the presence of ova, but his general health had greatly improved. This case of Chauffard seems to show that round worms, when present in large numbers, may give rise to serious symptoms, and Moosbrugger[10] believes that trichocephalus dispar, which is generally supposed to be a harmless parasite, may, when excessively numerous, be a cause of trouble. Anæmia and obstinate diarrhœa appear to be the most important symptoms, but colicky pains may be present. Mucus and, not infrequently, blood may also be present in the stools, the latter the consequence of intestinal ulceration. The diagnosis is made by microscopic examination of the fæces. The ova of the worm and Charcot's cystals found. The child attacked as a rule eventually gets well, but the disease lasts long. The author thinks that there is no radical treatment. Tonics should be used. Schwerdt[11] writes upon enteroptosis, his remarks being based upon a study of ninety-eight cases. Six occurred in men, eighty-nine in women. Associated with the altered position of the intestines, the stomach was displaced it sixty-nine cases, and the kidneys in eighty-six. One of the results of the lessened abdominal pressure is the accumulation of gas in the stomach and intestine. The development of poisonous products is thus facilitated. Dyspeptic symptoms are common, and later there may be palpitation, dyspnœa, headaches, and mental depression.

[1] Lancet, Mar. 14, p. 705. [2] Berlin Klin. Woch, Nos. 1 and 2, 1896. Med. Chronicle, Mar., 1896. [3] Wiener Med. Woch, No. 2, 1895. Med. Chronicle, Dec., 1895. [4] Brit. Med. J., Feb. 15, 1896. [5] Berlin Klin, Woch, 1895, No. 16; New Y. Med. J., Dec., 1895. [6] Wien. Klin. Woch. Oct., 1895; Brit. Med. J., Nov. 1895. [7] Internat. Med. Magazine, Feb, 1896. [8] Clin. Journal, Feb. 19, 1896. [9] Le Semaine Médicale, Feb., 1896; Med. Chron., Feb., 1896. [10] Münch Med. Woch, Nov. 1895; Brit. Med. J., Feb. 1, 1896. [11] Deut. Med. Woch, Jan., 1896; Brit, Med. J., Mar. 21, 1896.

